# Insulin resistance indices for predicting circadian syndrome: estimated glucose disposal rate as a protective indicator through non-linear dose-response in Chinese adults

**DOI:** 10.3389/fnut.2025.1718952

**Published:** 2026-01-12

**Authors:** Mengyi Zhang, Shenrui Xu, Yanying Guo

**Affiliations:** 1 Department of Endocrinology and Metabolism, People's Hospital of Xinjiang Uygur Autonomous Region, Xinjiang, China; 2 School of Biomedical Engineering, Guangzhou Medical University, Guangzhou, China; 3 Guangzhou National Laboratory, Guangzhou, China; 4 Xinjiang Clinical Research Center for Diabetes, Xinjiang, China

**Keywords:** CHARLS, circadian syndrome, estimated glucose disposal rate (eGDR), insulin resistance, population-based study

## Abstract

**Background:**

Circadian syndrome (CircS) extends metabolic syndrome by incorporating sleep and mood components, demonstrating superior predictive value for cardiovascular outcomes. Insulin resistance (IR) critically mediates the bidirectional metabolic-circadian disruption underlying CircS. While CircS affects nearly 40% of Chinese adults, the predictive utility of IR surrogate indices remains unexplored. This study evaluated the associations between six IR indices and both the prevalence and incidence of CircS among middle-aged and older Chinese adults.

**Methods:**

We examined associations between six IR indices—including estimated glucose disposal rate (eGDR), triglyceride-glucose-body mass index (TyG-BMI), and metabolic score for insulin resistance (METS-IR)—and CircS among 8,392 participants aged ≥45 years from the China Health and Retirement Longitudinal Study (CHARLS, 2011–2015) using cross-sectional and longitudinal designs. Logistic and Cox regression models assessed associations with covariate adjustment. Subgroup analyses by age and sex included interaction testing. Restricted cubic splines (RCS) and receiver operating characteristic (ROC) curves evaluated dose-response relationships and discriminatory performance.

**Results:**

CircS prevalence was 37.0% (3,101/8,392), with marked sex differences: women accounted for 69.0% of CircS cases vs. 44.0% of non-CircS cases (*P* < 0.001). Diabetes prevalence was 3.1-fold higher in CircS patients (11.2 vs. 3.6%, *P* < 0.001). All indices demonstrated excellent discrimination (AUC: 0.893–0.907). After full adjustment, eGDR showed inverse associations with CircS (OR = 0.381; HR = 0.602), while TyG-BMI, METS-IR, and AIP were positively associated with CircS risk in both analyses. RCS revealed varied dose-response patterns, with most indices showing non-linearity and AIP demonstrating a linear relationship. Sex and age significantly modified several index-CircS associations, with stronger associations observed among women and adults aged < 60 years.

**Conclusions:**

IR surrogate indices demonstrate strong associations with CircS. Notably, eGDR exhibited a protective effect with robust predictive value for incident CircS in longitudinal analysis. These readily calculable indices may be valuable for screening high-risk populations, particularly women and adults aged < 60 years.

## 1 Introduction

Metabolic syndrome (MetS) has served as a valuable clinical framework for identifying clusters of cardiometabolic risk factors that collectively increase the risk of cardiovascular disease and type 2 diabetes mellitus (T2DM), enabling clinicians to recognize co-occurring metabolic abnormalities that might otherwise be overlooked (1). While MetS remains clinically useful, emerging evidence highlights the need to consider circadian biology alongside traditional metabolic markers (2). The circadian system—orchestrated by the hypothalamic suprachiasmatic nucleus and synchronized with peripheral clocks in metabolic tissues—fundamentally regulates metabolism, hormone secretion, and sleep-wake cycles (3). Modern lifestyle factors including shift work, artificial light exposure, and irregular eating patterns disrupt this delicate system, contributing substantially to the global burden of non-communicable diseases and representing a promising therapeutic target (4–6). Recognizing this metabolic-circadian interconnection, circadian syndrome (CircS) extends the MetS framework by incorporating sleep disturbance and depression alongside traditional metabolic criteria (7). The expanded CircS model demonstrates superior predictive capacity for cardiovascular outcomes, with individuals meeting CircS but not MetS criteria exhibiting significantly elevated risk (8). This enhanced predictive value holds particular clinical relevance for diabetes management, as patients frequently experience sleep disorders and mood disturbances—both core CircS components that may further worsen metabolic control (9, 10).

Insulin resistance (IR) not only drives traditional metabolic abnormalities seen in MetS but also bidirectionally interacts with circadian rhythms—disrupted sleep patterns exacerbate IR, while IR itself impairs peripheral clock gene expression in metabolic tissues (11, 12). This reciprocal relationship positions IR as a critical mediator of CircS development. IR promotes both cardiovascular disease and T2DM through multiple pathways including systemic inflammation, endothelial dysfunction, and atherogenic dyslipidemia (13, 14). While the hyperinsulinemic-euglycemic clamp remains the gold standard for IR assessment, its clinical impracticality has prompted the development of surrogate indices using routine laboratory parameters (15). These include the estimated glucose disposal rate (eGDR); the triglyceride-glucose index (TyG) and its anthropometric derivatives (TyG-BMI); the metabolic score for IR (METS-IR); the Chinese visceral adiposity index (CVAI); and the atherogenic index of plasma (AIP), each capturing different aspects of IR pathophysiology. The ability of these readily available indices to predict CircS could provide clinicians with practical screening tools for identifying individuals at highest risk for this metabolic-circadian disorder.

However, the predictive utility of these IR indices for CircS remains unexplored in Chinese populations. Prior research has focused primarily on traditional cardiometabolic outcomes such as cardiovascular disease, stroke, and diabetes complications (16–18). However, these represent late-stage endpoints where irreversible damage has often occurred. The predictive utility of IR indices for CircS—a syndrome capturing earlier metabolic-circadian dysfunction before progression to severe cardiovascular events—remains unexplored in Chinese populations. Given that CircS represents a substantial public health burden in China and demonstrates superior cardiovascular predictive capacity compared to MetS alone, identifying optimal IR-based screening tools represents an urgent clinical priority. Fundamental questions remain unanswered: which IR index offers the most robust association with prevalent and incident CircS? Additionally, the potential for age- and sex-specific variations in index performance has not been systematically evaluated. Understanding these population-specific patterns is essential for developing targeted screening strategies that maximize early detection efficiency.

## 2 Methods

### 2.1 Data source and study population

CHARLS is a comprehensive longitudinal survey initiated in 2011, designed to examine the socioeconomic situation and health dynamics of individuals aged 45 and older in China. This study employed a multi-stage probability sampling method proportional to size to select 17,708 participants from 10,257 families throughout 150 counties in 28 provinces nationwide. Our work utilized data from CHARLS collected in 2011 and 2015 to conduct both cross-sectional and longitudinal analyses examining the relationship between IR surrogate indices and CircS risk. The CHARLS received approval from the Biomedical Ethics Review Committee at Peking University (IRB00001052-11015), and all participants provided informed consent.

The cross-sectional study's inclusion criteria were: (1) age ≥45 years; (2) complete data for CircS diagnosis. The exclusion criteria comprised: (1) participants lacking data on any of the six IR surrogate indices (eGDR, CVAI, TyG, TyG-BMI, METS-IR, and AIP); (2) participants without marital status information; (3) participants missing smoking status; (4) participants lacking drinking status; (5) participants without diabetes data; (6) participants missing low density lipoprotein cholesterol (LDL-C) concentration data; (7) participants lacking creatinine data.

**Figure 1:**
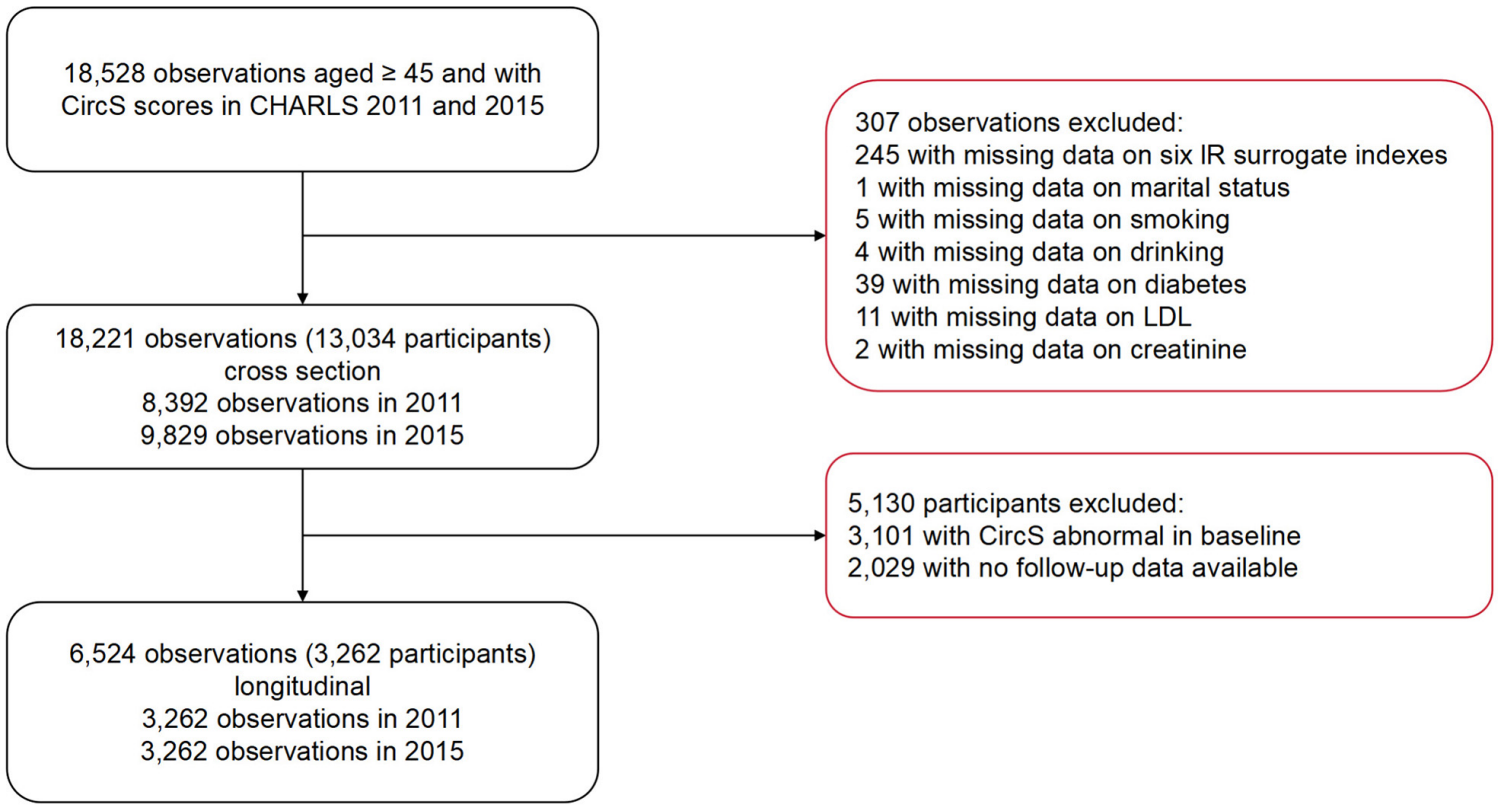
Participant selection flowchart.

The longitudinal study's inclusion criteria aligned with those of the cross-sectional study, while the exclusion criteria encompassed two additional items: (1) participants diagnosed with CircS at baseline and (2) participants with incomplete follow-up data. The participant selection flowchart is shown in Figure 1.

### 2.2 Exposure: six IR surrogate indices

Six indices were selected as proxies of IR, including: eGDR, CVAI, TyG, TyG-BMI, METS-IR, and AIP. The specific calculation formula of each index was:


$$\begin{array}{l} eGDR=21.158-[0.09\times waist\;circumference\;(cm)]\\ \;-[3.407\times hypertension\;(yes=1,\;no=0)]\\ \;-[0.551\times HbA1c\;(\%)] \end{array}$$

$$BMI=weight(kg)/height{\left(m\right)}^{2}$$

$$\begin{array}{l} CVAI_{\left(male\right)}=-267.93+[0.68\times age(years)]\\ \;+[0.03\times BMI(kg/m^{2})]+[4.00\times waist\;circumference\;(cm)]\\ \;+\{22.00\times \log_{10}[triglycerides\;(mmol/L)]\}-[16.32\times high\\ \;-density\;lipoprotein\;cholesterol\;(mmol/L)] \end{array}$$

$$\begin{array}{l} CVAI_{\left(female\right)}=-187.32+\left[1.71\times age(years)\right]\\ \;+[4.23\times BMI(kg/m^{2})]+[1.12\times waist\;circumference\;(cm)]\\ \;+\{39.76\times \log_{10}[triglycerides\;(mmol/L)]\}\\ \;-[11.66\times high-density\;lipoprotein\;cholesterol\;(mmol/L)] \end{array}$$

$$\begin{array}{l} TyG=ln[triglycerides(mg/dl)\times fasting\;plasma\;glucose\\ \;(mg/dl)/2] \end{array}$$

$$TyG-BMI=TyG\times BMI(kg/m^{2})$$

$$\begin{array}{l} METS-IR=ln[2\times fasting\;plasma\;glucose\;(mg/dl)\\ \;+triglycerides\;(mg/dl)]\times BMI(kg/m^{2})/ln[high\\ \;-density\;lipoprotein\;cholesterol(mg/dl)] \end{array}$$

$$\begin{array}{l} AIP=\;\log[triglycerides(mg/dl)/high\;-\;density\;lipoprotein\\ \;cholesterol\;(mg/dl)] \end{array}$$

### 2.3 Outcome: CircS

The concept of CircS is derived from MetS, and its diagnosis includes the following seven components: short sleep duration (< 6 h daily), central obesity [male: waist circumference (WC) ≥85 cm; female: WC ≥80 cm], elevated triglyceride (TG) levels [≥150 mg/dl (1.7 mmol/L)], reduced high-density lipoprotein cholesterol (HDL-C) [male: < 40 mg/dl (1.0 mmol/L); female: < 50 mg/dl (1.3 mmol/L)], hypertension (systolic blood pressure ≥130 mmHg and/or diastolic blood pressure ≥85 mmHg), hyperglycemia (≥100 mg/dl), and depression. The ten-item Center for Epidemiologic Studies Depression Scale-10 (CESD-10) was employed to evaluate depressive symptoms. The overall score ranges from 0 to 30 points, with elevated scores signifying more severe depressive symptoms. Individuals fulfilling four or more components are classified as possessing CircS; otherwise, they are deemed to lack CircS (7).

### 2.4 Covariates

Covariates in this study included: (1) demographic information: age (< 60 and ≥60); gender (female and male); education levels (less than junior high school education, high school and vocational training, higher education), and marital status (unmarried or separated, marriage or cohabitation); (2) health status and functioning: smoking (yes and no), drinking (yes and no), sleep duration, BMI, WC, diabetes, and hypertension; (3) laboratory test data: total cholesterol (TC), HDL-C, LDL-C, creatinine, uric acid, blood urea nitrogen (BUN), and C-reactive protein (CRP).

### 2.5 Robustness verification with modified CircS definitions

To assess the robustness of associations independent of component overlap between IR indices and CircS diagnostic criteria, sensitivity analyses were conducted with modified CircS definitions. For each IR index, CircS components sharing constituent variables with that index were excluded, and modified CircS was defined as meeting ≥3 of the remaining non-overlapping criteria (Supplementary Table S2). Primary analyses were then repeated using these modified definitions.

### 2.6 Statistical analysis

Descriptive analysis utilized mean ± standard deviation (SD) for continuous variables and percentages (%) for categorical variables. The Wilcoxon test (continuous variables) and the Chi-square test (categorical variables) were applied to examine the differences between the groups. In the cross-sectional study, the association between IR surrogate indices and CircS risk was evaluated by the logistic regression models. Three models were developed: Model 1 was unadjusted; Model 2 adjusted for age, gender, education levels, marital status, smoking, drinking, BMI, and WC; and Model 3 adjusted for Model 2 plus sleep duration, diabetes, hypertension, TC, HDL-C, LDL-C, creatinine, uric acid, BUN, and CRP. Restricted cubic splines (RCS) analysis was conducted to determine linear or non-linear relationships between IR surrogate indices and CircS risk. Additionally, subgroup analysis was carried out to investigate the consistency of associations across subgroups (age and gender). Receiver operating characteristic (ROC) curves were generated to evaluate the predictive effectiveness of IR surrogate indices for CircS risk. The Cox regression models in the longitudinal investigation assessed the relationship between IR surrogate measures and CircS risk, with remaining procedures aligned with the cross-sectional study.

All analyses were performed using R software (version 4.4.3). A *P*-value below 0.05 was deemed statistically significant.

## 3 Results

### 3.1 Baseline characteristics

After exclusions, 8,392 participants were enrolled in our study, including 3,101 participants diagnosed with CircS and 5,291 participants without CircS (Table 1). The CircS population had a higher proportion of females compared to males (69.0 vs. 31.0%), while the non-CircS group displayed an inverse tendency (44.0 vs. 56.0%). Both groups were predominantly characterized by low educational attainment (less than junior high school: CircS 91.9%, non-CircS 89.8%) and married/cohabiting status (CircS 81.5%, non-CircS 85.0%). Smoking and drinking rates were lower in the CircS group compared to the non-CircS group. Compared to participants without CircS, CircS patients exhibited higher CVAI, TyG, TyG-BMI, METS-IR, AIP, BMI, WC, TC, LDL-C, uric acid, and CRP levels, with a higher proportion of hypertension and diabetes, while eGDR, sleep duration, HDL-C, creatinine, and BUN were lower. All baseline characteristics showed significant differences between CircS and non-CircS groups (*P* < 0.05).

**Table 1:** Baseline demographic, clinical, and metabolic characteristics of study participants stratified by CircS status (*n* = 8,392).

**Variables**	**Overall**	**Non-CircS**	**CircS**	***P*-value**
	8,392	5,291	3,101	
CircS status (mean, SD)	0.37 (0.48)	0.00 (0.00)	1.00 (0.00)	< 0.001
eGDR (mean, SD)	9.62 (2.06)	10.26 (1.71)	8.52 (2.14)	< 0.001
CVAI (mean, SD)	−528.06 (268.38)	−628.87 (252.00)	−356.07 (199.17)	< 0.001
TyG (mean, SD)	8.69 (0.66)	8.43 (0.50)	9.13 (0.66)	< 0.001
TyG-BMI (mean, SD)	205.47 (40.98)	190.30 (32.42)	231.35 (41.11)	< 0.001
METS-IR (mean, SD)	35.74 (8.37)	32.49 (6.16)	41.27 (8.74)	< 0.001
AIP (mean, SD)	0.82 (0.76)	0.52 (0.59)	1.34 (0.74)	< 0.001
Age (mean, SD)	59.75 (9.11)	59.38 (9.23)	60.37 (8.88)	< 0.001
**Gender (%)**				
Female	4,469 (53.3)	2,328 (44.0)	2,141 (69.0)	< 0.001
Male	3,923 (46.7)	2,963 (56.0)	960 (31.0)	
**Education level (%)**				
Less than junior high school education	7,602 (90.6)	4,752 (89.8)	2,850 (91.9)	0.004
High school and vocational training	699 (8.3)	481 (9.1)	218 (7.0)	
Higher education	91 (1.1)	58 (1.1)	33 (1.1)	
**Marital status (%)**				
Unmarried or separated	1,366 (16.3)	792 (15.0)	574 (18.5)	< 0.001
Marriage or cohabitation	7,026 (83.7)	4,499 (85.0)	2,527 (81.5)	
**Smoking (%)**				
No	5,835 (69.5)	3,361 (63.5)	2,474 (79.8)	< 0.001
Yes	2,557 (30.5)	1,930 (36.5)	627 (20.2)	
**Drinking (%)**				
No	5,689 (67.8)	3,289 (62.2)	2,400 (77.4)	< 0.001
Yes	2,703 (32.2)	2,002 (37.8)	701 (22.6)	
BMI (kg/m^2^, mean (SD))	23.56 (3.83)	22.55 (3.44)	25.29 (3.85)	< 0.001
Waist circumference (cm, mean, SD)	85.63 (9.95)	82.63 (9.01)	90.74 (9.38)	< 0.001
Sleep night (hours, mean, SD)	6.34 (1.91)	6.67 (1.75)	5.79 (2.03)	< 0.001
Total cholesterol (mg/dl, mean, SD)	193.78 (38.35)	189.88 (36.23)	200.43 (40.86)	< 0.001
HDL cholesterol (mg/dl, mean, SD)	51.08 (15.25)	55.72 (14.58)	43.17 (12.93)	< 0.001
LDL cholesterol (mg/dl, mean, SD)	116.91 (35.16)	115.98 (32.73)	118.50 (38.93)	0.002
Creatinine (mg/dl, mean, SD)	0.79 (0.24)	0.79 (0.21)	0.78 (0.28)	0.001
Uric acid (mg/dl, mean, SD)	4.47 (1.26)	4.42 (1.24)	4.55 (1.29)	< 0.001
Blood urea nitrogen (mg/dl, mean, SD)	15.75 (4.58)	15.97 (4.63)	15.37 (4.47)	< 0.001
C-reactive protein (mg/dl, mean, SD)	2.75 (7.64)	2.56 (7.90)	3.08 (7.15)	0.002
**Hypertension (%)**				
No	6,109 (72.8)	4,342 (82.1)	1,767 (57.0)	< 0.001
Yes	2,283 (27.2)	949 (17.9)	1,334 (43.0)	
**Diabetes (%)**				
No	7,854 (93.6)	5,100 (96.4)	2,754 (88.8)	< 0.001
Yes	538 (6.4)	191 (3.6)	347 (11.2)	

### 3.2 Associations between IR surrogate indices and CircS risk

Tables 2, 3 present the correlations between IR surrogate indicators and CircS risk. The cross-sectional study indicated that after full adjustment (Model 3), eGDR was a protective factor for CircS occurrence (OR = 0.381, 95% CI: 0.277–0.524), while AIP, METS-IR, TyG-BMI, and TyG were risk factors for CircS occurrence (AIP: OR = 4.089, 95% CI: 3.399–4.918; METS-IR: OR = 61.517, 95% CI: 40.52–93.395; TyG-BMI: OR = 25.057, 95% CI: 18.007–34.865; TyG: OR = 3.515, 95% CI: 3.089–4.000; Table 2). However, the association between CVAI and CircS was not significant (*P* = 0.548).

**Table 2:** Cross-sectional associations between IR indices and CircS: logistic regression analysis.

**Variable**		**Model 1**	**Model 2**	**Model 3**
eGDR	OR(95% CI)	0.404 (0.384–0.426)	0.561 (0.525–0.599)	0.381 (0.277–0.524)
	*P* value	4.887E-264	1.498E-65	2.791E-09
CVAI	OR(95% CI)	5.968 (5.467–6.516)	4.533 (4.103–5.009)	0.835 (0.464–1.503)
	*P* value	< 1E-300	1.008E-193	5.477E-01
TyG	OR(95% CI)	4.347 (4.046–4.669)	4.001 (3.703–4.323)	3.515 (3.089–4.000)
	*P* value	< 1E-300	4.135E-270	5.792E-81
TyG-BMI	OR(95% CI)	3.701 (3.466–3.952)	36.332 (29.680–44.473)	25.057 (18.007–34.865)
	*P* value	< 1E-300	1.029E-265	2.041E-81
METS-IR	OR(95% CI)	4.325 (4.029–4.642)	23.642 (19.876–28.120)	61.517 (40.520–93.395)
	*P* value	< 1E-300	1.113E-279	2.581E-83
AIP	OR(95% CI)	4.41 (4.108–4.735)	4.302 (3.974–4.658)	4.089 (3.399–4.918)
	*P* value	< 1E-300	5.424E-284	1.974E-50

**Table 3:** Longitudinal associations between IR surrogate indices and incident CircS: Cox proportional hazards regression models.

**Variable**		**Model 1**	**Model 2**	**Model 3**
eGDR	HR(95% CI)	0.685 (0.642–0.731)	0.758 (0.694–0.828)	0.602 (0.523–0.693)
	*P* value	2.389E-30	7.575E-10	1.677E-12
CVAI	HR(95% CI)	1.998 (1.800–2.218)	1.563 (1.382–1.768)	4.480 (2.744–7.314)
	*P* value	1.408E-38	1.211E-12	2.023E-09
TyG	HR(95% CI)	1.332 (1.239–1.433)	1.317 (1.215–1.427)	1.097 (0.972–1.237)
	*P* value	1.143E-14	2.170E-11	1.338E-01
TyG-BMI	HR(95% CI)	1.332 (1.276–1.391)	2.024 (1.617–2.532)	1.189 (1.121–1.260)
	*P* value	1.296E-38	7.308E-10	6.611E-09
METS-IR	HR(95% CI)	1.303 (1.251–1.357)	1.873 (1.587–2.211)	1.181 (1.112–1.254)
	*P* value	4.011E-37	1.146E-13	6.531E-08
AIP	HR(95% CI)	1.398 (1.298–1.505)	1.411 (1.300–1.531)	1.232 (1.027–1.478)
	*P* value	7.040E-19	1.623E-16	2.449E-02

The longitudinal study demonstrated that following adjustment (Model 3), eGDR was negatively associated with CircS risk (HR = 0.602, 95% CI: 0.523–0.693), while CVAI, TyG-BMI, METS-IR, and AIP showed opposite trends (CVAI: HR = 4.480, 95% CI: 2.744–7.314; TyG-BMI: HR = 1.189, 95% CI: 1.121–1.260; METS-IR: HR = 1.181, 95% CI: 1.112–1.254; AIP: HR = 1.232, 95% CI: 1.027–1.478; Table 3). However, the correlation between TyG and CircS was not marked (*P* = 0.134).

Therefore, except for CVAI (non-significant in cross-sectional analysis) and TyG (non-significant in longitudinal analysis), the remaining indices achieved significance in both analyses.

These findings were further explored through conservative sensitivity analyses using modified CircS definitions (excluding overlapping components; Supplementary Tables S3, S4). In unadjusted models, all six indices maintained significant associations with modified CircS (cross-sectional *P*: 10^−15^ to 10^−236^; longitudinal *P*: 10^−5^ to 10^−29^). After full adjustment, TyG, TyG-BMI, and AIP retained cross-sectional significance (*P* ≤ 0.002), while eGDR (HR = 0.578, *P* = 0.004) retained longitudinal significance.

### 3.3 Dose-response relationships between IR surrogate indices and CircS risk

**Figure 2:**
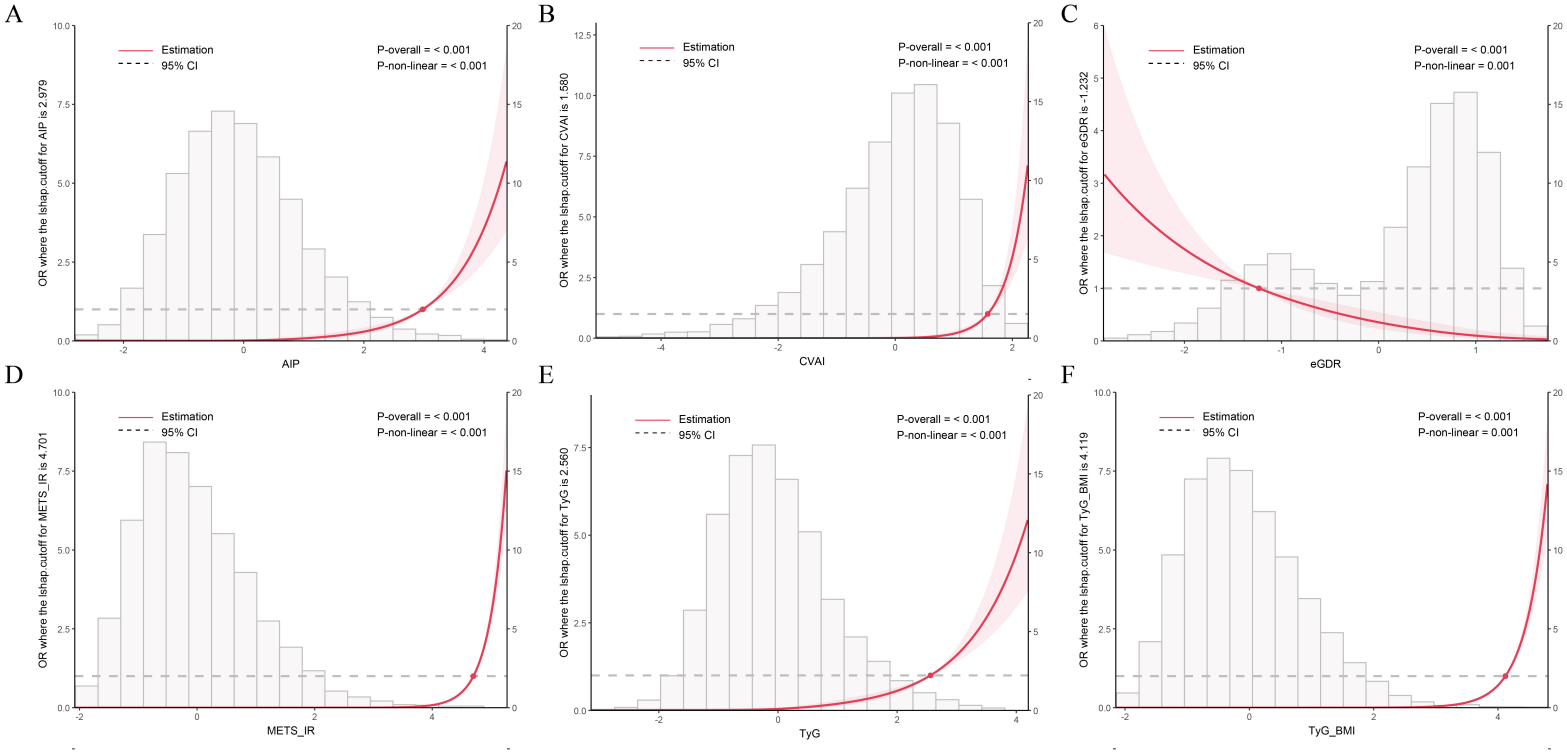
Dose-response relationships between IR surrogate indices and CircS prevalence. Restricted cubic spline analysis from cross-sectional CHARLS data. **(A)** AIP. **(B)** CVAI. **(C)** eGDR. **(D)** METS-IR. **(E)** TyG. **(F)** TyG-BMI. Solid lines represent odds ratios with 95% confidence intervals (dashed lines).

Restricted cubic spline (RCS) analysis was conducted to examine linear or non-linear relationships between IR surrogate indices and CircS risk. All IR surrogate indices showed significant non-linear associations with CircS risk in the cross-sectional analysis (*P*_−overall_ < 0.001, *P*_−non − linear_ < 0.001; Figures 2A–F).

**Figure 3:**
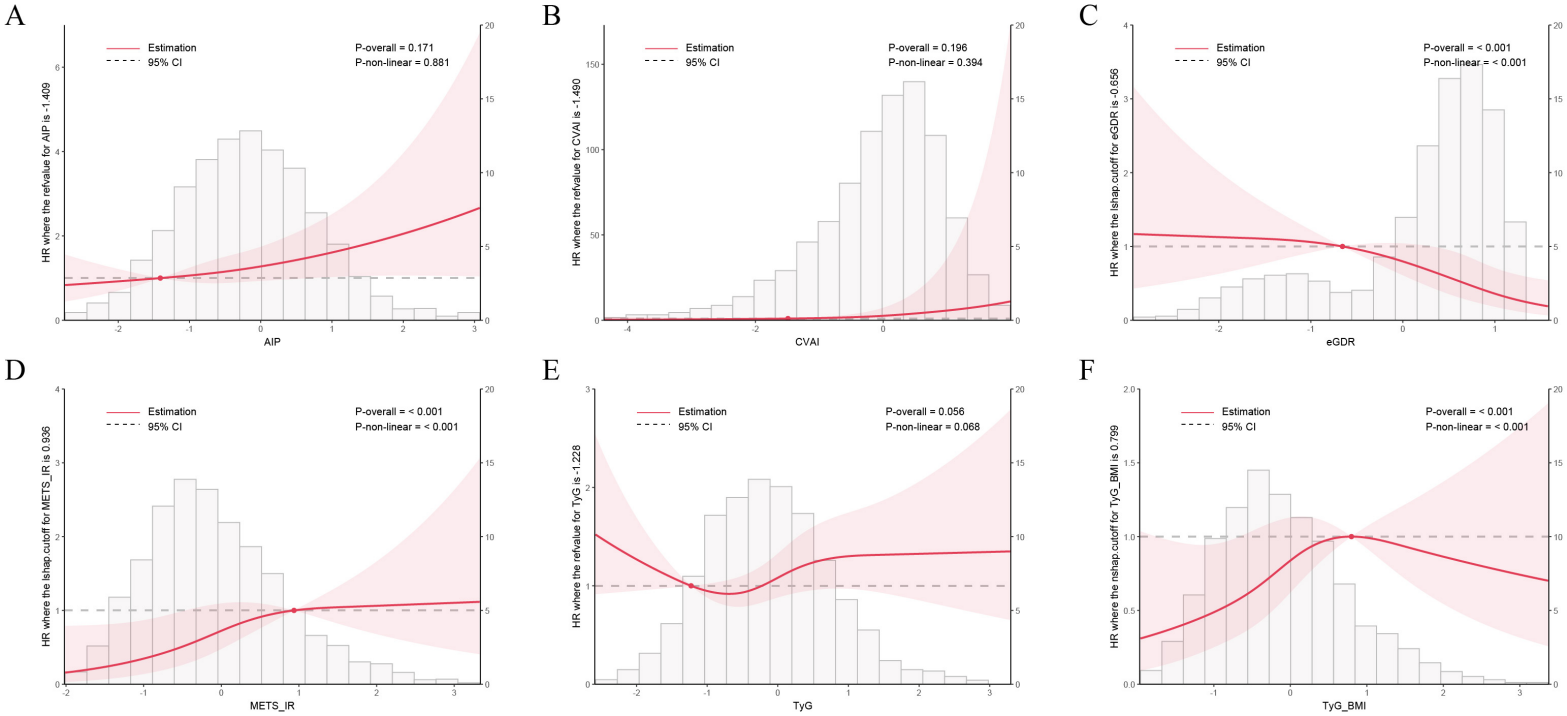
Dose-response relationships between IR surrogate indices and incident CircS risk. RCS analysis from longitudinal CHARLS data. **(A)** AIP. **(B)** CVAI. **(C)** eGDR. **(D)** METS-IR. **(E)** TyG. **(F)** TyG-BMI. Solid lines represent hazard ratios with 95% confidence intervals (dashed lines).

In the longitudinal analysis, only eGDR, TyG-BMI, and METS-IR exhibited significant non-linear associations with CircS risk (*P*_−overall_ < 0.001, *P*_−non − linear_ < 0.001), while AIP showed a linear relationship (Figures 3A–F).

### 3.4 Subgroup analysis

**Figure 4:**
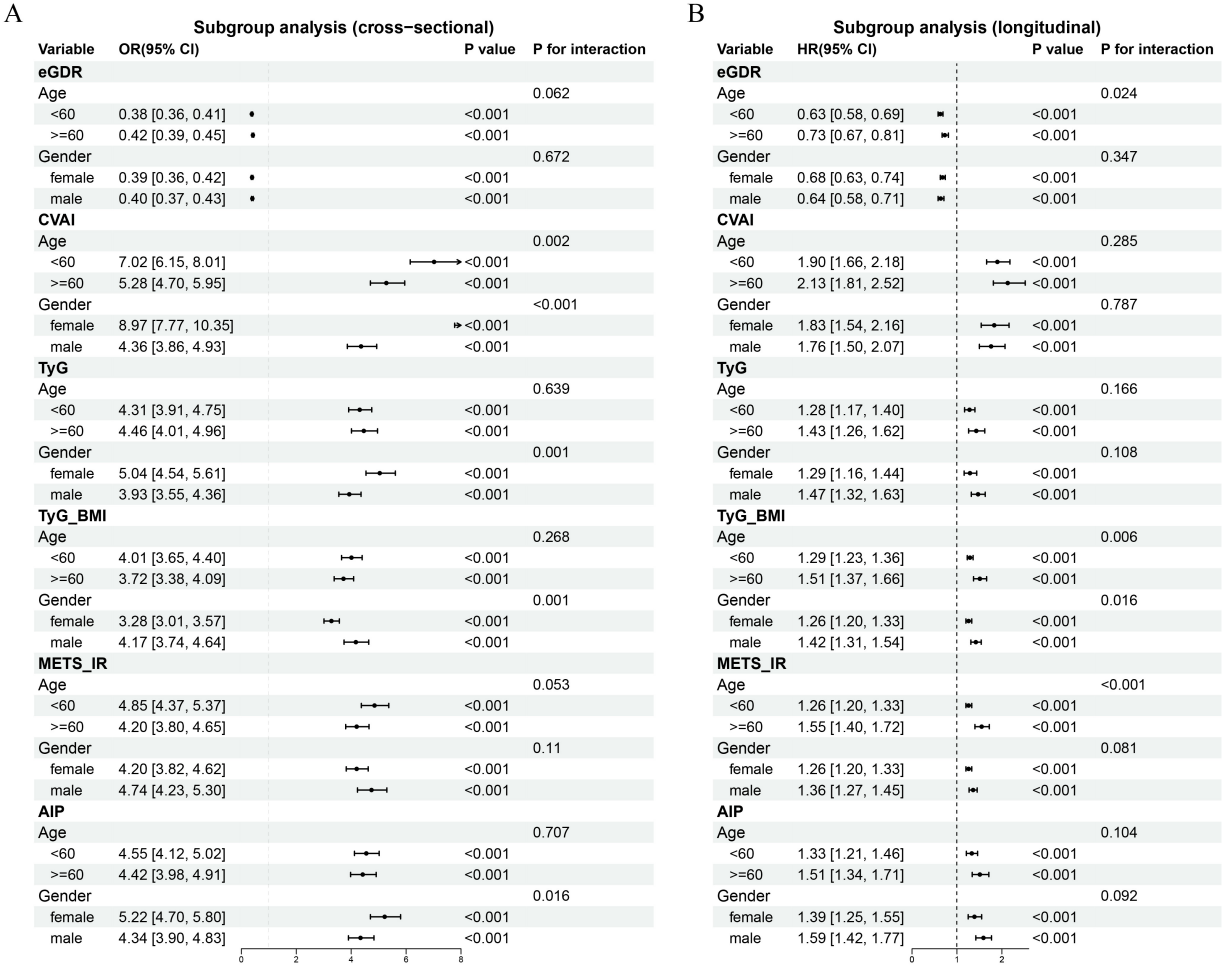
Subgroup analyses of associations between IR surrogate indices and CircS risk stratified by age and sex. **(A)** Cross-sectional associations showing odds ratios with 95% confidence intervals. **(B)** Longitudinal associations showing hazard ratios with 95% confidence intervals. Age groups: < 60 vs. ≥60 years; gender groups: female vs. male. All models adjusted for potential confounders.

We conducted subgroup and interaction analyses based on age and gender to examine the relationship between IR surrogate indices and CircS risk across various demographic parameters. Results demonstrated that in the cross-sectional study, an increase in eGDR was consistently associated with a decrease in the prevalence of CircS, and the remaining indices showed opposite trends (Figure 4A). Interaction analysis revealed that age and gender moderated the relationship between CVAI and CircS risk (interaction: age: *P* = 0.002; gender: *P* < 0.001), and gender moderated the relationships between TyG and CircS risk (interaction: *P* = 0.001), TyG-BMI and CircS risk (interaction: *P* = 0.001), and AIP and CircS risk (interaction: *P* = 0.016).

In the longitudinal study, all IR surrogate indices exhibited consistent correlations with incident CircS (Figure 4B). Notable interactions were detected: age and gender moderated the association between TyG-BMI and CircS (age interaction: *P* = 0.006; gender interaction: *P* = 0.016), and age moderated the associations between eGDR and CircS (*P* = 0.024) as well as between METS-IR and CircS (*P* < 0.001).

### 3.5 Discriminative performance of IR surrogate indices for CircS

**Figure 5:**
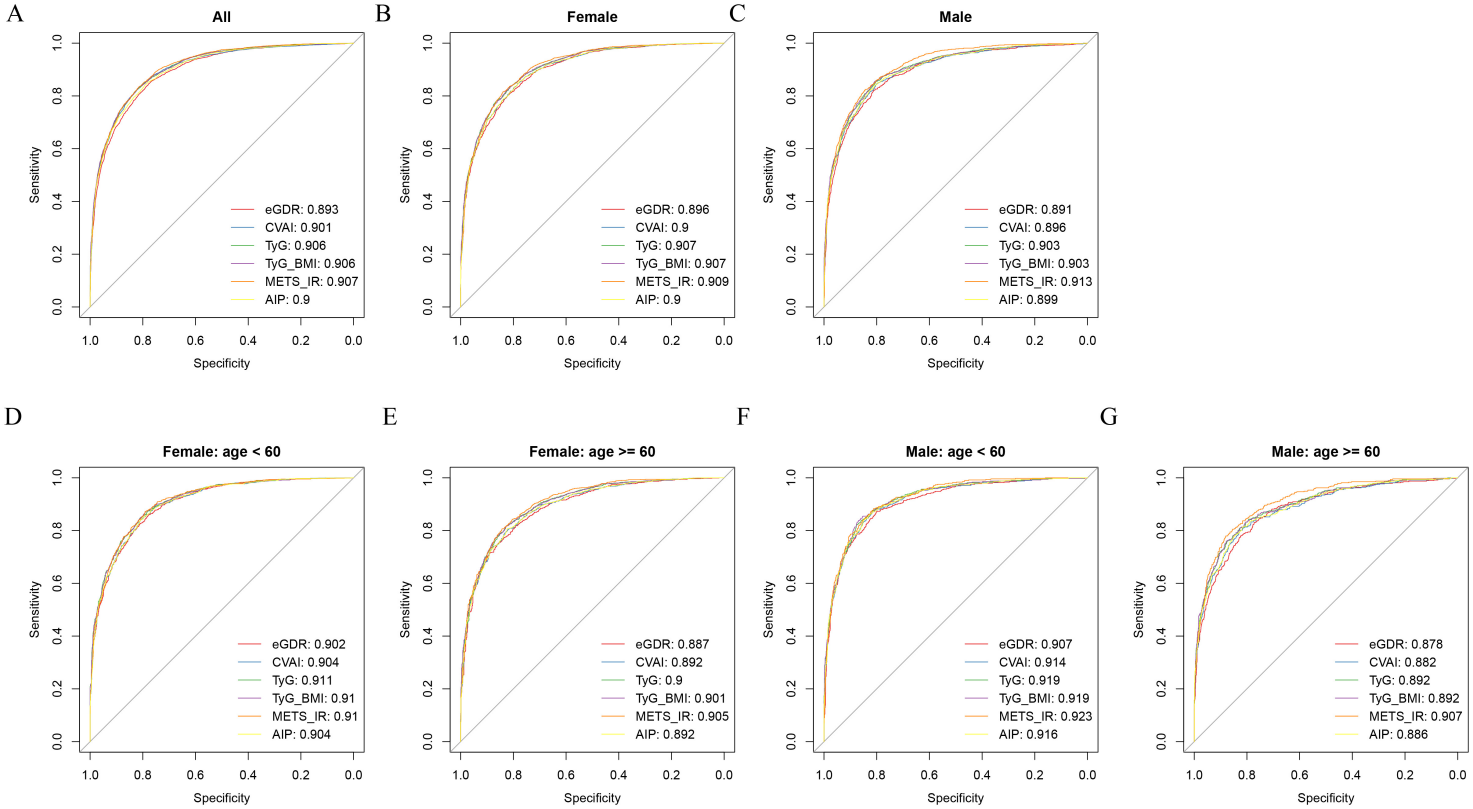
ROC curves evaluating the discriminatory performance of IR surrogate indices for CircS prediction across demographic subgroups. **(A)** Overall population. **(B)** Female. **(C)** Male. **(D)** Female aged < 60 years. **(E)** Female aged ≥60 years. **(F)** Male aged < 60 years. **(G)** Male aged ≥60 years. AUC values with 95% confidence intervals are displayed for each index.

ROC curves were generated to evaluate the discriminative ability of IR surrogate indicators for CircS. The results indicated that eGDR achieved an AUC of 0.893, while other indices all exceeded 0.9 (detailed data in Supplementary Table S1; Figure 5A). In both sexes, all IR surrogate indices exhibited strong discriminative ability for CircS (detailed data in Supplementary Table S1; Figures 5B, C). Furthermore, except for METS-IR, the other indices demonstrated superior discriminative performance in the female group relative to the male group (Figures 5B, C). When further stratified by age (< 60 vs ≥60 years), both sexes demonstrated better discrimination in the younger age group (Figures 5D–G).

## 4 Discussion

### 4.1 Principal findings and comparative performance of IR indices

This study systematically evaluated six IR surrogate indices for identifying prevalent and incident CircS in 8,392 Chinese adults aged ≥45 years. While all indices demonstrated comparable discriminative ability (AUC: 0.893–0.907), their performance diverged markedly between cross-sectional and longitudinal analyses, with single-component indices showing particularly limited longitudinal predictive value. Our findings complement previous studies that established associations between IR indices and traditional cardiometabolic outcomes (16–18). Whereas, prior work focused on IR surrogates' utility for predicting complications of diabetes or cardiovascular events, we demonstrate their specific application to CircS—a metabolic-circadian phenotype that uniquely integrates sleep and mood disturbances with metabolic dysfunction. This application to metabolic-circadian identification and prediction offers clinicians practical tools for screening patients at risk for a syndrome particularly relevant to modern lifestyle-related health challenges.

Among the evaluated indices, eGDR emerged as particularly noteworthy, demonstrating both excellent discriminative capacity and unique protective characteristics (OR = 0.381, HR = 0.602). By integrating three critical dimensions—glycemic control (HbA1c), vascular health (hypertension status), and central adiposity (waist circumference)—eGDR captures a comprehensive insulin sensitivity phenotype that extends beyond simple glucose metabolism. Our findings align with recent work (19), which demonstrated eGDR's inverse association with MetS prevalence and mortality. The robust inverse association between eGDR and CircS suggests a potential mechanistic link: preserved insulin sensitivity may protect against circadian disruption through maintenance of peripheral clock gene expression in metabolically active tissues. This hypothesis gains support from clinical observations in type 1 diabetes mellitus, where eGDR selectively identifies pathological sleep disorders requiring intervention while remaining stable in individuals with physiological sleep variations (20). This selective responsiveness—distinguishing pathological from normal sleep variability—represents a critical advantage for CircS screening, as it minimizes false positives that might arise from benign sleep pattern variations common in aging populations.

In contrast to eGDR's protective effects, TyG-BMI and METS-IR showed strong associations with prevalent CircS (OR = 25.057 and 61.517, respectively) and maintained significant, though more modest, associations with incident CircS (HR = 1.189 and 1.181, respectively). The substantial attenuation from cross-sectional ORs to longitudinal HRs merits explanation. Component overlap between IR indices and CircS criteria—while partly reflecting shared pathophysiology—may contribute to the larger cross-sectional effect sizes where exposure and outcome are measured simultaneously. The longitudinal design, with temporal separation between baseline exposure and incident outcome, reduces this contribution. Additionally, TyG-BMI remained significant in both analyses, whereas TyG lost longitudinal significance (HR=1.097, *P* = 0.134), underscoring the added value of incorporating adiposity measures. This finding aligns with recent evidence, which demonstrated that composite IR indices (TyG-WHtR, TyG-WC) maintained predictive value while the standalone TyG index failed to predict mortality (21). Together, these observations reveal fundamental principle: incorporating adiposity measures transforms glycemic-lipid indices into robust predictors by capturing the full spectrum of metabolic dysfunction—including adipose tissue distribution and ectopic lipid accumulation that drive progressive IR (22). We also noted that, CVAI exhibited marked reduction after full adjustment in cross-sectional analysis, while the other five indices maintained consistent effect directions and significance in fully adjusted models. CVAI's instability likely relates to its having the highest number of constituent variables and the greatest overlap with adjusted covariates.

### 4.2 Sensitivity analyses confirming association robustness

To rigorously assess the independence of these associations from component overlap, we conducted sensitivity analyses using modified CircS definitions that excluded overlapping components—a deliberately conservative approach. In unadjusted models, all indices maintained highly significant associations (*P* < 10^−5^), indicating predictive capacity beyond shared variance. In fully adjusted models, several indices retained significance: TyG, TyG-BMI, and AIP in cross-sectional analysis (*P* < 0.01), and eGDR (HR = 0.578, *P* = 0.004) in longitudinal analysis. The attenuation of certain associations likely reflects over-adjustment: overlapping components were first excluded from the outcome definition, then related metabolic parameters were additionally controlled as covariates—a dual removal that inevitably diminishes residual signal. These sensitivity findings reinforce our primary analyses—particularly for eGDR, which demonstrated robust associations even under this conservative framework. eGDR's sustained longitudinal significance strengthens confidence in its reliability for long-term risk stratification.

### 4.3 Diabetes-CircS interconnection and clinical implementation

A striking finding was the three-fold higher diabetes prevalence among CircS patients compared to non-CircS individuals (11.2 vs. 3.6%, *P* < 0.001), confirming a strong metabolic-circadian interconnection. This observation carries important clinical implications. First, patients with diabetes warrant prioritized CircS screening given their elevated risk. Second, the substantial overlap suggests shared pathophysiological pathways involving IR, explaining why IR indices effectively predict both conditions. Third, the high comorbidity burden indicates that isolated management of either condition may yield suboptimal outcomes. These findings align with emerging evidence of bidirectional causality—diabetes may precipitate CircS through metabolic disruption, while CircS can exacerbate glycemic dysregulation (23, 24). For diabetes clinics, implementing IR-based CircS screening could involve: (1) automated calculation of eGDR at each visit using routine HbA1c and anthropometric data; (2) flagging patients whose IR indices deviate significantly from population norms for comprehensive CircS evaluation; (3) intensifying metabolic control in those with confirmed CircS, recognizing the need for integrated metabolic-circadian management beyond standard diabetes care. This approach transforms CircS from an underrecognized comorbidity to an actionable clinical target within existing diabetes care pathways.

### 4.4 Age-dependent effect modifications and clinical implications

Our subgroup analyses revealed age-dependent effect modifications with important implications for clinical implementation. The protective effect of eGDR was more pronounced in adults < 60 years (HR = 0.63) compared to those ≥60 years (HR = 0.74; *P*_−interaction_ = 0.024), identifying middle age as a critical intervention window. This age-related attenuation likely reflects the natural decline in metabolic flexibility with aging—older adults have reduced ability to switch between burning fats and carbohydrates for energy due to decreased mitochondrial function (25). Conversely, risk indices (TyG-BMI, METS-IR) demonstrated stronger associations with CircS in older adults in longitudinal analysis (TyG-BMI: *P*_−interaction_ = 0.006; METS-IR: *P*_−interaction_ < 0.001). Aging disrupts circadian rhythms through reduced NAD+/SIRT1 signaling, impairing the clock's metabolic responsiveness (26). This mechanistic insight may explain why IR indices show stronger associations with CircS in older adults—their compromised circadian-metabolic interface cannot adequately buffer metabolic disruptions. These age-specific modifications suggest tailored intervention strategies: middle-aged adults with preserved metabolic flexibility may benefit from aggressive IR management to prevent CircS development, while older adults require integrated approaches addressing both metabolic dysfunction and circadian desynchronization.

### 4.5 Female predominance and postmenopausal vulnerability

The female predominance in CircS (69.0%) reflects complex endocrine-metabolic interactions beyond ascertainment bias. In our cohort (mean age 59.75 years), postmenopausal estrogen decline disrupts metabolic-circadian coupling. Estrogen normally enhances insulin sensitivity via ERα-PI3K-Akt-Foxo1 signaling, suppressing hepatic gluconeogenesis while promoting peripheral glucose uptake (27, 28). It also maintains pancreatic β-cell function by promoting insulin secretion and preventing apoptosis (29). Its postmenopausal loss therefore promotes dual metabolic insults: peripheral IR and β-cell dysfunction. Importantly, IR indices showed better discriminative ability for CircS in women than in men, with optimal performance in those aged < 60 years—suggesting that screening during the perimenopausal transition, before metabolic deterioration becomes fully established, may maximize detection efficiency. These findings suggest that women in midlife and beyond as represent a priority population for IR-based screening.

### 4.6 Dose-response patterns of metabolic indices

Longitudinal RCS analyses revealed distinct dose-response patterns among IR indices, with eGDR and AIP demonstrating a meaningful contrast. Specifically, eGDR showed a significant non-linear protective association with CircS risk: HR remained relatively stable at lower eGDR values, but declined steeply as eGDR increased—suggesting that greater insulin sensitivity is associated with more pronounced risk reduction. In contrast, AIP exhibited a linear positive association, with CircS risk increasing proportionally across its entire range without apparent thresholds. This difference in patterns may reflect distinct metabolic characteristics: eGDR integrates multidimensional information including glycemic control, blood pressure, and central adiposity, whereas AIP primarily reflects lipid metabolism status.

### 4.7 Strengths and Limitations

This study's strengths include the use of a nationally representative cohort with rigorous sampling methods, ensuring generalizability to Chinese middle-aged and elderly adults, while the integration of cross-sectional and longitudinal designs strengthens causal inference beyond what either approach could achieve alone. The comprehensive evaluation of six IR indices, compared to the 2–3 typically examined in prior studies, enables robust comparison of predictive efficacy, and the application of restricted cubic splines alongside subgroup interaction analyses provides nuanced insights into non-linear relationships and population heterogeneity. Despite these methodological advantages, several limitations warrant consideration. First, component overlap between IR indices and CircS diagnostic criteria represents an inherent methodological challenge. The high cross-sectional ORs may reflect both biological associations and shared components. Sensitivity analyses using modified CircS definitions supported the robustness of eGDR. However, these modified definitions deviate from the original CircS framework, and the primary analyses remain the core basis for this study. Second, the absence of fasting insulin data in CHARLS precluded calculation of HOMA-IR and direct comparison with this established IR measure. This limitation reflects the reality of community-based research—fasting insulin is not routinely measured in primary care settings. Third, the focus on adults ≥45 years restricts generalizability to younger populations—although CircS is increasingly prevalent among younger adults, research in this group remains limited.

## 5 Conclusion

This study demonstrates that most IR surrogate indices are significantly associated with CircS and exhibit excellent discriminatory performance in Chinese adults aged ≥45 years. Notably, eGDR emerges as the only protective indicator, showing consistent inverse associations with both prevalent and incident CircS. Even after excluding components overlapping with CircS diagnostic criteria, eGDR retains robust predictive value in longitudinal analysis. Subgroup analyses further reveal stronger associations of these indices with CircS among women and adults aged < 60 years. Given its reliable predictive performance, inherent protective implication, and ease of calculation using routine clinical parameters, eGDR holds particular promise as a practical screening tool for CircS, especially in high-risk populations such as postmenopausal women and patients with diabetes.

## Data Availability

Publicly available datasets were analyzed in this study. This data can be found here: the China Health and Retirement Longitudinal Study (CHARLS) repository. Access to these data can be obtained by registering and submitting a request through the official CHARLS website at https://charls.pku.edu.cn.
