# Supplementary material for: Insulin resistance indices for predicting circadian syndrome: estimated glucose disposal rate as a protective indicator through non-linear dose-response in Chinese adults
Source: Front Nutr. 2026 Jan 12;12:1718952. doi: 10.3389/fnut.2025.1718952 (PMC12832305; doi:10.3389/fnut.2025.1718952)
Supplement: Supplementary file 1 [file Data_Sheet_1.docx]

Supplementary Material

**TABLE S1. ROC curves of IR surrogate indexes and CircS risk in different populations.**

| **Group** | **Variable** | **AUC (95% CI)** | ***P*** | ***P* for comparison** | **Optimal cutoff value** | **Sensitivity** | **Specificity** | **Youden index** |
| --- | --- | --- | --- | --- | --- | --- | --- | --- |
| All | eGDR | 0.893 (0.886, 0.900) | < 0.001 | Reference | -0.748 | 0.846 | 0.778 | 0.624 |
|  | CVAI | 0.901 (0.895, 0.908) | < 0.001 | < 0.001 | -0.653 | 0.847 | 0.801 | 0.647 |
|  | TyG | 0.906 (0.899, 0.912) | < 0.001 | < 0.001 | -0.452 | 0.814 | 0.832 | 0.647 |
|  | TyG-BMI | 0.906 (0.899, 0.912) | < 0.001 | < 0.001 | -0.608 | 0.836 | 0.813 | 0.649 |
|  | METS-IR | 0.907 (0.901, 0.913) | < 0.001 | < 0.001 | -0.678 | 0.833 | 0.816 | 0.649 |
|  | AIP | 0.900 (0.893, 0.906) | < 0.001 | < 0.001 | -0.686 | 0.841 | 0.792 | 0.633 |
| Male | eGDR | 0.891 (0.879, 0.903) | < 0.001 | Reference | -1.142 | 0.821 | 0.812 | 0.633 |
|  | CVAI | 0.896 (0.884, 0.908) | < 0.001 | 0.017 | -1.221 | 0.848 | 0.798 | 0.646 |
|  | TyG | 0.903 (0.892, 0.914) | < 0.001 | < 0.001 | -1.264 | 0.855 | 0.802 | 0.657 |
|  | TyG-BMI | 0.903 (0.892, 0.915) | < 0.001 | < 0.001 | -1.260 | 0.854 | 0.808 | 0.662 |
|  | METS-IR | 0.913 (0.903, 0.923) | < 0.001 | < 0.001 | -1.138 | 0.822 | 0.846 | 0.668 |
|  | AIP | 0.899 (0.888, 0.910) | < 0.001 | 0.001 | -1.172 | 0.840 | 0.810 | 0.650 |
| Female | eGDR | 0.896 (0.887, 0.905) | < 0.001 | Reference | -0.123 | 0.810 | 0.813 | 0.623 |
|  | CVAI | 0.900 (0.891, 0.908) | < 0.001 | 0.0130 | -0.301 | 0.847 | 0.784 | 0.631 |
|  | TyG | 0.907 (0.899, 0.916) | < 0.001 | < 0.001 | -0.184 | 0.836 | 0.811 | 0.647 |
|  | TyG-BMI | 0.907 (0.899, 0.915) | < 0.001 | < 0.001 | -0.180 | 0.833 | 0.814 | 0.647 |
|  | METS-IR | 0.909 (0.900, 0.917) | < 0.001 | < 0.001 | -0.091 | 0.816 | 0.840 | 0.655 |
|  | AIP | 0.900 (0.891, 0.909) | < 0.001 | 0.006 | -0.365 | 0.854 | 0.777 | 0.631 |

**TABLE S2. Modified CircS definitions after excluding overlapping components with each IR index.** Checkmarks indicate the CircS components retained for modified CircS diagnosis after excluding variables overlapping with each respective IR index. Modified CircS was diagnosed when individuals met the specified number of remaining criteria.

| **IR Index** | **Waist circumference** | **Triglyceride** | **HDL-c** | **Blood pressure** | **Blood glucose** | **Sleep** | **CES-D** | **Case_num(≥)** |
| --- | --- | --- | --- | --- | --- | --- | --- | --- |
| eGDR |  | √ | √ |  |  | √ | √ | 3 |
| CVAI |  |  |  | √ | √ | √ | √ | 3 |
| TyG | √ |  | √ | √ |  | √ | √ | 3 |
| TyG_BMI | √ |  | √ | √ |  | √ | √ | 3 |
| METS_IR | √ |  |  | √ |  | √ | √ | 3 |
| AIP | √ |  |  | √ | √ | √ | √ | 3 |

**TABLE S3. Cross-sectional associations between IR indices and modified CircS (excluding overlapping components).**

| **Variable** |  | **Model 1** | **Model 2** | **Model 3** |
| --- | --- | --- | --- | --- |
| eGDR | OR(95% CI) | 0.710(0.670-0.751) | 0.845(0.781-0.914) | 0.754(0.531-1.069) |
|  | *P*-value | 6.720E-32 | 2.793E-05 | 1.128E-01 |
| CVAI | OR(95% CI) | 1.242(1.178-1.310) | 1.070(0.999-1.145) | 1.027(0.682-1.546) |
|  | *P*-value | 1.511E-15 | 5.298E-02 | 8.989E-01 |
| TyG | OR(95% CI) | 2.002(1.903-2.107) | 1.659(1.569-1.755) | 1.181(1.062-1.312) |
|  | *P*-value | 7.534E-157 | 5.356E-70 | 2.134E-03 |
| TyG_BMI | OR(95% CI) | 2.562(2.422-2.710) | 3.614(3.131-4.172) | 1.548(1.187-2.020) |
|  | *P*-value | 5.137E-236 | 6.426E-69 | 1.254E-03 |
| METS_IR | OR(95% CI) | 1.543(1.467-1.624) | 1.107(1.002-1.223) | 0.825(0.638-1.067) |
|  | *P*-value | 1.139E-62 | 4.637E-02 | 1.424E-01 |
| AIP | OR(95% CI) | 1.615(1.541-1.692) | 1.305(1.238-1.375) | 1.484(1.291-1.706) |
|  | *P*-value | 3.315E-90 | 2.703E-23 | 2.804E-08 |

**TABLE S4. Longitudinal associations between IR indices and incident modified CircS (excluding overlapping components).**

| **Variable** |  | **Model 1** | **Model 2** | **Model 3** |
| --- | --- | --- | --- | --- |
| eGDR | HR(95% CI) | 0.774(0.713-0.841) | 0.865(0.772-0.968) | 0.578(0.397-0.843) |
|  | *P*-value | 1.379E-09 | 1.184E-02 | 4.336E-03 |
| CVAI | HR(95% CI) | 1.214(1.109-1.330) | 1.019(0.908-1.142) | 0.690(0.381-1.251) |
|  | *P*-value | 2.788E-05 | 7.512E-01 | 2.219E-01 |
| TyG | HR(95% CI) | 1.179(1.101-1.263) | 1.094(1.014-1.179) | 1.038(0.913-1.179) |
|  | *P*-value | 2.825E-06 | 2.010E-02 | 5.713E-01 |
| TyG_BMI | HR(95% CI) | 1.322(1.26-1.388) | 1.216(1.01-1.465) | 1.035(0.76-1.410) |
|  | *P*-value | 1.438E-29 | 3.918E-02 | 8.253E-01 |
| METS_IR | HR(95% CI) | 1.287(1.216-1.361) | 1.122(0.976-1.289) | 0.808(0.565-1.155) |
|  | *P*-value | 2.321E-18 | 1.064E-01 | 2.419E-01 |
| AIP | HR(95% CI) | 1.206(1.124-1.293) | 1.102(1.02-1.19) | 1.098(0.914-1.319) |
|  | *P*-value | 1.699E-07 | 1.332E-02 | 3.164E-01 |
